# Serum naturally occurring anti-TDP-43 auto-antibodies are increased in amyotrophic lateral sclerosis

**DOI:** 10.1038/s41598-021-81599-5

**Published:** 2021-01-21

**Authors:** Elisa Conti, Gessica Sala, Susanna Diamanti, Marco Casati, Christian Lunetta, Francesca Gerardi, Claudia Tarlarini, Lorena Mosca, Nilo Riva, Yuri Falzone, Massimo Filippi, Ildebrando Appollonio, Carlo Ferrarese, Lucio Tremolizzo

**Affiliations:** 1 Lab of Neurobiology, School of Medicine and Surgery and Milan Center for Neuroscience, University of Milano-Bicocca, Monza, Italy; 2 Neurology Unit, “San Gerardo” Hospital, ASST Monza, Monza, Italy; 3 Laboratory of Chemical and Clinical Analyses, “San Gerardo” Hospital, ASST Monza, Monza, Italy; 4 NEuroMuscular Omnicentre (NEMO), Fondazione Serena Onlus, Milano, Italy; 5 Medical Genetics Unit, ASST Grande Ospedale Metropolitano Niguarda, Milano, Italy; 6 Experimental Neuropathology Unit, Division of Neuroscience, IRCCS “San Raffaele” Scientific Institute, Milano, Italy; 7 Neurology Unit, Neurorehabilitation Unit, Neurophysiology Service, Division of Neuroscience, IRCCS “San Raffaele” Scientific Institute, Milano, Italy; 8 Neuroimaging Research Unit, Division of Neuroscience, IRCCS “San Raffaele” Scientific Institute, Milano, Italy; 9 Vita-Salute San Raffaele University, Milano, Italy; 10 Room 2043, U8 building, Via Cadore 48, 20900 Monza, MB Italy

**Keywords:** Neuroscience, Biomarkers

## Abstract

Amyotrophic Lateral Sclerosis (ALS) patients express significant clinical heterogeneity that often hinders a correct diagnostic definition. Intracellular deposition of TDP-43, a protein involved in RNA metabolism characterizes the pathology. Interestingly, this protein can be detected in serum, wherein cognate naturally-occurring auto-antibodies (anti-TDP-43 NAb) might be also present, albeit they have never been documented before. In this exploratory study, we quantified the levels of both anti-TDP-43 NAb and TDP-43 protein as putative accessible markers for improving the ALS diagnostic process by using ELISA in *N* = 70 ALS patients (*N* = 4 carrying TARDBP mutations), *N* = 40 age-comparable healthy controls (CTRL), *N* = 20 motor neuron disease mimics (MN-m), *N* = 20 Alzheimer’s disease (AD) and *N* = 15 frontotemporal lobar degeneration (FTLD) patients. Anti-TDP-43 NAb were found to be significantly increased in ALS patients compared to all the other groups (*p* < 0.001). On the other hand, the distribution of serum levels of TDP-43 protein was highly variable among the various groups. Levels were increased in ALS patients, albeit the highest values were detected in MN-m patients. NAb and protein serum levels failed to correlate. For the first time, we report that serum anti-TDP-43 NAb are detectable in human serum of both healthy controls and patients affected by a variety of neurodegenerative disorders; furthermore, their levels are increased in ALS patients, representing a potentially interesting trait *core* marker of this disease. Further studies are needed to clarify the exact role of the NAb. This information might be extremely useful for paving the way toward targeting TDP-43 by immunotherapy in ALS.

## Introduction

Amyotrophic lateral sclerosis is a relentless neurodegenerative disorder that preferentially affects motor neurons. TAR DNA-binding protein 43 (TDP-43) is a protein coded by the *TARDBP* gene whose mutations are known to cause ALS. TDP-43 inclusions are typically found in affected neurons, even in non-genetically mediated cases^1^.

Remarkably, intracellular TDP-43 inclusions have also been detected in peripheral blood mononuclear cells (PBMC) of ALS patients^2–4^, raising suspicion of a potential functional derangement of immune cells in a disorder for which the contribution of neuroinflammation has been repeatedly reported^5^. Abnormal inflammatory responses are directly sustained by protein aggregates (collectively known as DAMPs, i.e., danger associated molecular patterns^6^ resulting from both impaired proteostasis and progressive exhaustion of adaptive immunity along lifetime, a condition known as *inflammaging*^7^.

Presumably, the adaptive immune response against TDP-43 inclusions contemplates the production of polyclonal antibodies, as already demonstrated in DAMPs associated to a variety of neurodegenerative disorders, such as beta-amyloid for Alzheimer’s disease (AD) and alpha-synuclein for Parkinson’s disease^8,9^.

Although the presence of naturally occurring anti-TDP-43 auto-antibodies (NAbs) could be hypothesized, it is unknown whether ALS patients express a different quantity of NAb as compared to healthy controls and whether they play a pathogenic role in ALS onset and progression. Notably, the hypothesis that a decrease of anti-DAMP serum NAb could be related to an increased protein aggregation has already been formulated to explain neurodegeneration^10,11^.

On the other hand, besides intracellular aggregates, serum (and CSF) levels of soluble TDP-43 have been previously reported to be moderately increased in ALS patients^12^, although the diagnostic value of this observation is scarce^13^. One may hypothesize that increased levels of soluble TDP-43 in serum could contribute to an increased production of specific NAb.

To verify these hypotheses, we assessed the presence of anti-TDP-43 NAb and TDP-43 protein in the serum of n = 165 subjects, represented by both patients affected by ALS, frontotemporal lobar degeneration (FTLD), AD and other motor neuron disorder mimics (MN-m), and healthy controls. Then, we quantified the serum levels of anti-TDP-43 NAb and TDP-43 protein and we investigated the putative clinical correlate of these serum *core* biomarkers of TDP-43 pathology.

## Materials and methods

### Patient recruitment and characterization

The study was approved by San Gerardo Hospital ethical committee (protocol: Anti Abeta AD 21/04/2011), patients and controls voluntarily agreed to participate in the study and informed consent was obtained from all subjects. Patients and controls gave their consent for publication. *N* = 70 ALS outpatients diagnosed according to El Escorial criteria were recruited at the NEMO center (Milano, Italy); among them, *N* = 4 ALS patients were carrier of a *TARDBP* mutation (TDP43+ : c.881G>T (p.Gly294Val) [*N* = 1]; c.1144G>A (p.Ala382Thr) [*N* = 2]; c.1147A>G (p.Ile383Val) [*N* = 1]). None of the other patients were carrying mutations in other major ALS causative genes (*SOD1*, *FUS*, *C9ORF72*), with the exception of one patient carrying a *SOD1* mutation (p.Ile150Thr). For this preliminary study, full-blown dementia was suspected based on the clinical interview with the ALS patient and the respective caregiver, and eventually defined according to DSM-V criteria. *N* = 40 sex- and age-comparable healthy controls (CTRL) were recruited as well. Furthermore, we included the following consecutive outpatients: *N* = 20 typical Alzheimer’s disease (AD) patients according to IWG-2 criteria (always including confirmed low CSF beta-amyloid and high total- and phospho-tau levels)^14^; *N* = 15 FTLD patients according to current criteria for bvFTD^15^, with a CSF profile that excluded AD and without clinical evidence of motor neuron disease (MND); *N* = 20 MND mimics (MN-m, including: *N* = 6 diabetic motor polyneuropathies, *N* = 7 idiopathic motor polyneuropathies, *N* = 7 cervical or lumbar radiculopathies). FTLD patients were age- and sex-comparable to ALS, whereas both AD and MN-m patients were significantly older (~ 10 years) than ALS patients and more representative in females (+ 20%). Patients with dementia were recruited if either (a) a legal representative was available or (b) if they were able to express informed consent (mostly mild-severity cases; this competence was confirmed by the MacArthur Competence Assessment Tool).

Exclusion criteria for all groups were the use of steroid or immunosuppressive drugs, recent infection or surgery, cancer or autoimmune/inflammatory diseases. See Table 1 for the demographic and clinical characteristics of the recruited sample.

**Table 1:** Clinical and demographic characteristics of the recruited sample.

Group	ALS	TDP43+	CTRL	AD	FTLD	MN-m
*N*	66	4	40	20	15	20
M (%)	45 (68.1%)	1 (25%)	25 (62.5%)	9 (45%)	10 (66.7%)	9 (45%)
Age, *years*	63.6 ± 10.1 (37–79)	57.0 ± 8.8 (50–70)	64.5 ± 11.2 (39–82)	74.7 ± 3.3 (67–80)	65.6 ± 7.3 (56–77)	72.3 ± 13.8 (51–88)
Onset, *Spinal/Bulbar*	53/13	4/0	–	–	15 bvFTD	–
Disease duration, *months since estimated onset*	24.5 ± 15.1 (5–62)	42.3 ± 39.9 (9–97)	–	34.8 ± 22.3 (12–84)	31.9 ± 18.1 (6–60)	33.9 ± 15.9 (10–60)
Functional scale	*ALSFRS-R* 32.3 ± 8.8 (8–45)	*ALSFRS-R* 33.2 ± 1.8 (32–36)	–	*MMSE* 19.2 ± 5.9 (6–25)	*FTLD_modified CDR SoB* 13.0 ± 4.7 (3.5–16.0)	na
DPI^a^	0.82 ± 0.65 (0.10–2.69)	0.69 ± 0.54 (0.16–1.32)	–	0.36 ± 0.20 (0.13–0.80)	na	na
BMI	23.1 ± 3.1 (17.2–31.5)	26.3 ± 4.8 (20.2–31.9)	na	na	na	na
Riluzole, *yes* (%) 50 mg b.i.d	54 (81%)	3 (75%)	–	0	0	0
EN, *yes* (%)	5 (7.5%)	0	–	0	0	0
NIV, *yes* (%)	9 (13.6%)	1 (25%)	–	0	0	0
Dementia, *yes* (%)	2 (3%)	1 (25%)	0	20 (100%)	15 (100%)	0

*AD* Alzheimer’s disease, *ALSFRS-R* ALS Functional Rating Scale-revised version, *BMI* body mass index, *CTRL* controls, *DPI* disease progression index, *EN* enteral nutrition, *MMSE* Mini-Mental State Examination, *na* not available, *NIV* non-invasive ventilation, *TDP43* + ALS patients carrying a pathogenic *TARDBP* mutation. Data are shown as mean ± SD.

^a^The DPI was calculated as [48-ALSFRS-R score]/disease duration (months) for ALS patients and [30-MMSE score]/disease duration (months) for AD patients.

### Serum anti-TDP-43 NAb and TDP-43 assessment

Blood samples (5 ml) were collected after overnight (O/N) fasting. Serum was obtained by centrifugation (2800 g 20 min) and stored at − 80 °C until blind-coded assay. The concentration of anti-TDP-43 NAb was determined by indirect ELISA, specifically developed for the target. Different setting regarding protein coating concentration (100, 50, 25 and 10 µg/ml), blocking buffer (traditional or protein free), calibration curve range (3000 to 6.25 ng/ml) and sample dilution (from undiluted to 1:300) were tested. Eventually, 96-well plates (Greiner Bio One) were coated at 4 °C O/N with 25 µg/ml human TDP-43 full length protein (Abcam) in 50 mM carbonate buffer (pH 9.6), blocked with SmartBlock (CANDOR Bioscience) (200 µl/well) for 60 min at room temperature (RT) with shaking and washed 3 times with PBS. A standard curve was generated using an affinity-purified mouse (monoclonal) anti-TDP-43 antibody (Santa Cruz Biotechnology), at different concentrations, ranging from 400 to 6.25 ng/ml (serial dilutions). Blank wells were included to subtract out the non-specific binding of serum antibody. Coated plates were incubated at 4 °C O/N with standard curve and serum samples (1:200 dilution). After washing with PBS-T (Tween 20, 0.05%), plates were incubated for 2 h with HRP conjugated goat anti-mouse IgG antibodies (Sigma-Aldrich) 1:12,000 for the standard curve and HRP conjugated goat anti-human IgG antibodies 1:10,000 (Sigma–Aldrich) for samples at RT with shaking. Then, plates were washed with PBS-T and incubated for 10 min with TMB (Sigma–Aldrich); after adding the stop solution, the absorbance at 450 nm was read. The concentration of serum NAb was determined from the standard curve and read in triplicate.

*Precision:* two samples of known concentration were tested 20 times on a single plate to assess intra-assay precision and in 20 separate assays to assess inter-assay precision. Inter- and intra-assay variability was lower than 5 and 10% respectively. *Sensitivity:* the lower limit of quantification (LLOQ) was calculated based on the signal and SD of 16 blank samples (10 SDs above the mean blank sample). LLOQ of the assay is 34.5 ng/ml.

Total IgG serum content was measured by an automated immunoturbidimetric analysis on Modular P analyzer (Roche Diagnostics). Serum samples were also analyzed for the presence of TDP43 protein by a commercially available ELISA kit (LSBio) following manufacturer’s instructions.

All methods were carried out in accordance with relevant guidelines and regulations.

### Statistical analysis

Statistical analysis was performed using Graph Prism 7.04 (GraphPad Software, La Jolla, CA). Data are shown as mean ± SD. ANOVA, followed by Tukey post hoc test, was used to assess differences among the groups. Two-tailed Pearson's *R*-test was used for correlative analyses.

## Results

**Figure 1:**
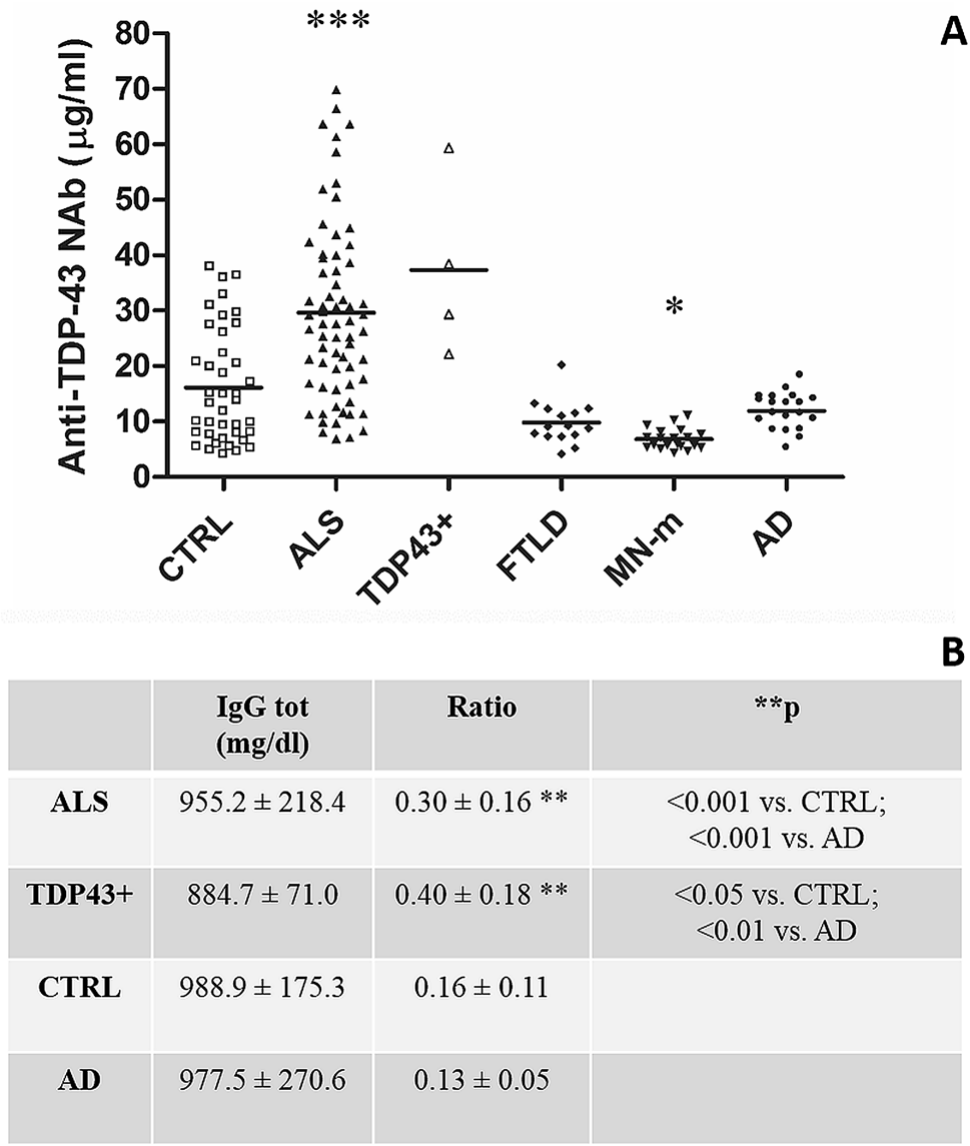
(**A**) Serum anti-TDP-43 NAb are increased in ALS patients. ANOVA *p* < 0.0001, followed by Tukey post hoc test ***p < 0.001 ALS vs. all the other groups, *p < 0.01 MN-m vs. CTRL and AD (**B**) the anti-TDP-43 Nab/total IgG ratio showed similar results, and total IgG were similar among recruited subjects. ANOVA *p* < 0.001, followed by **Tukey post hoc test.

Anti-TDP-43 NAb were detected in the serum of all subjects; serum levels were higher (+ 85%) in ALS patients compared to CTRLs, and they were two-to-threefold increased in ASL patients compared to AD and FTLD patients. Notably, serum anti-TDP-43 NAb levels were about fourfold lower in MN-m than in ALS patients (and besides twofold lower in MN-m patients than in CTRLs; Fig. 1A). These results did not change substantially when considering the ratio to total serum IgG (not available for FTLD and MN-m, see Fig. 1B). The *N* = 4 TDP-43 + ALS patients presented NAb levels and ratio similar to those expressed by TDP-43 negative ALS patients (Fig. 1).

**Figure 2:**
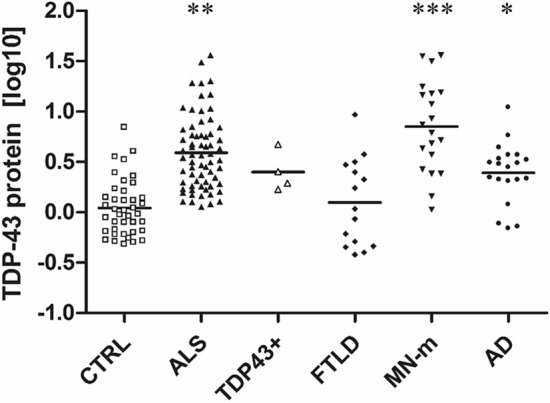
Serum TDP-43 soluble levels are increased in ALS and MN-m patients. ANOVA *p* = 0.0001, followed by Tukey post hoc test **p < 0.001 ALS vs. CTRL and FTLD, ***p < 0.0001 MN-m vs. all the other group with the exception of *p* < 0.05 vs. ALS, *p < 0.05 AD vs. CTRL.

Serum TDP-43 levels resulted to be widely variable, with a distribution characterized by a pronounced right skewness; log10 transformed values are shown in Fig. 2 (for the original data see Supplementary Fig. 1). ALS patients displayed higher serum levels of TDP-43 (about fivefold) than those detected in CTRLs and FTLD patients but lower levels than those of MN-m (about 50%) patients. In fact, the MN-m group exhibited the most accentuated dispersion and was -on average- the group with the highest serum levels of TDP-43. Finally, AD patients displayed slightly increased values with respect to CTRLs (Fig. 2).

No correlation between TDP-43 and cognate NAb serum levels in any group (p = 0.14 for ALS) was found. A positive correlation between TDP-43 serum levels and the disease progression index (DPI, see Table 1; *R* = 0.41 *p* = 0.001) was found. No correlations between biological data and any other clinical and demographic variable were found.

## Discussion

We report for the first time the presence of immunoreactivity suggestive for the existence of anti-TDP-43 NAb in human serum samples, whose levels resulted to be significantly high in a consistent number of ALS patients, apparently regardless of the presence of *TARDBP* mutations (only four carriers were included, with a wide distribution of clinical expression parameters). Serum TDP-43 protein levels resulted to be increased as well, confirming the results of previous observations^12^; however, their distribution showed a positive asymmetry, plausibly making it less useful for the purpose of developing a diagnostic biomarker, as previously noted by other Authors^13,16^.

The disease specificity of the NAb increase was here magnified by the inclusion not only of healthy controls, but also of patients affected by a variety of neurodegenerative disorders, such as AD—wherein an altered TDP-43 proteostasis is not expected, although this consideration has been recently challenged, as reported inthe case of “*LATE*”^17^)—and FTLD that could, at least in some cases, share a TDP-43 pathology. The group of MN-m allowed to further underline the potential clinical usefulness of this finding, since MN-m patients displayed very high (and variable) serum levels of TDP-43 protein, despite very low levels of cognate NAb. Certainly, sample size at this time is limited, at least for the pathological control groups, but this is a preliminary report and we are currently recruiting for a larger confirmatory study. In particular, more data regarding ALS clinical phenotype are going to be collected, in order to analyze further any putative relationship of blood-based TDP-43 biomarkers with disease signs, progression, and with the degree of cognitive impairment (not included at the moment).

In fact, many points need to be clarified. The NAb increase in the ALS cohort was apparently unrelated to clinical and demographic parameters and, albeit target-specific (see the IgG ratio), it did not correlate with the serum levels of the cognate protein. As a trait/core marker of TDP-43 pathology, the NAb could be extremely useful for the differential diagnosis, but the absolute confirmation of their presence and the understanding of their exact physiological role need to be further investigated; future studies should also include the analysis of the CSF compartment.

Concerning the physiological role of the NAb, it could be hypothesized that NAb might prevent excessive TDP-43 accumulation, in accordance with several recent experimental evidences^18–20^. Against this interpretation, one may point out the fact that MN-m displayed a strong dissociation between NAb and protein levels. Further data is necessary to draw any conclusions, since pronounced inflammatory conditions, such as some ALS mimics, exhibit different modalities of immune system involvement as compared to neurodegenerative disorders like ALS. Similar issues have been deeply debated in other neurological disorders; for example, anti-beta-amyloid NAb are typically found in both AD and in healthy controls^8,21^, and they are also detectable in patients with the inflammatory form of cerebral amyloid angiopathy^22^.

Besides some methodological issues related to the acid-dissociation of immunocomplexes before NAb assessment—that introduced a variable that has not been completely understood—some evidences suggest an increased immunoreactivity towards toxic Abeta species^23^. These findings fit well with the hypothesis that the generation of toxic protein affecting the immune-surveillance may apply to the TDP-43 pathology and they could be the ground for designing novel probative experiments. Meanwhile, a variety of specific NAb have been described in the neurodegenerative field, raising more questions than answers regarding both methodology and significance of these findings^24,25^.

A possible limit of this exploratory study consisted in having tested serum immunoreactivity (NAb) against the full-length form of TDP-43, since phosphorylated (and other) species could potentially be more toxic and, therefore, elicit a more robust immune response. Nevertheless, we should not forget that we are marking a polyclonal immunoreactivity against TDP-43 that presumably would have been captured also in case we had designed our assay using the phosphorylated full-length form of the same protein (work in progress). In any case, uncertainty remains regarding the toxic potential of modified or truncated species of TDP-43, such that the choice of a potentially “meaningful” NAb a priori is challenging. Moreover, future studies will be also necessary to discriminate which immunoglobulin subspecies are involved.

In spite of these limits, we are planning to test serum anti-TDP-43 NAb levels in a larger cohort of carefully phenotyped ALS patients to better elucidate the real usefulness of this marker for ALS diagnosis and clinical follow-up. This data would also be potentially useful to develop anti-TDP-43 immunotherapies for ALS patients^26^.

## Supplementary Information

Supplementary Figure 1.
