# Supplementary material for: Serum naturally occurring anti-TDP-43 auto-antibodies are increased in amyotrophic lateral sclerosis
Source: Sci Rep. 2021 Jan 21;11:1978. doi: 10.1038/s41598-021-81599-5 (PMC7820419; doi:10.1038/s41598-021-81599-5)

**Serum Naturally Occurring Anti-TDP-43 Auto-Antibodies Are Increased in Amyotrophic Lateral Sclerosis**

Elisa Conti,^1^ Gessica Sala,^1^ Susanna Diamanti,^1,2^ Marco Casati,^3^ Christian Lunetta,^4^ Francesca Gerardi,^4^ Claudia Tarlarini,^4^ Lorena Mosca,^5^ Nilo Riva,^6,7^ Yuri Falzone,^6,7^ Massimo Filippi,^7,8,9^ Ildebrando Appollonio,^1,2^ Carlo Ferrarese,^1,2^ Lucio Tremolizzo,^1,2^

^1^Lab of Neurobiology, University of Milano-Bicocca and Milan Center for Neuroscience; ^2^Neurology Unit, and ^3^Laboratory of Chemical and Clinical Analyses, “San Gerardo” hospital, ASST Monza, Monza; ^4^NEuroMuscular Omnicentre (NEMO), Fondazione Serena Onlus, Milano; ^5^Medical Genetics Unit, ASST Grande Ospedale Metropolitano Niguarda, Milano; ^6^Experimental Neuropathology Unit, ^7^Neurology Unit, Neurorehabilitation Unit, Neurophysiology Service, ^8^Neuroimaging Research Unit, Division of Neuroscience, IRCCS “San Raffaele” Scientific Institute, Milano; ^9^Vita-Salute San Raffaele University, Milano, Italy

**Word count:** 2379 **Abstract:** 247 words

**References:** 26 **Figures/Tables:** 2/1

**Journal:** Scientific Reports

**Please send correspondence to:**

Lucio Tremolizzo, M.D., Ph.D.

School of Medicine and Surgery - University of Milano-Bicocca

U8, Via Cadore 48 – 20900 Monza MB - Italy

Phone: +39-02.6448.8128

E-mail: [lucio.tremolizzo@unimib.it](mailto:lucio.tremolizzo@unimib.it)

**Supplementary Figure 1**

Serum TDP-43 soluble levels are increased in ALS and MN-m patients: raw data.


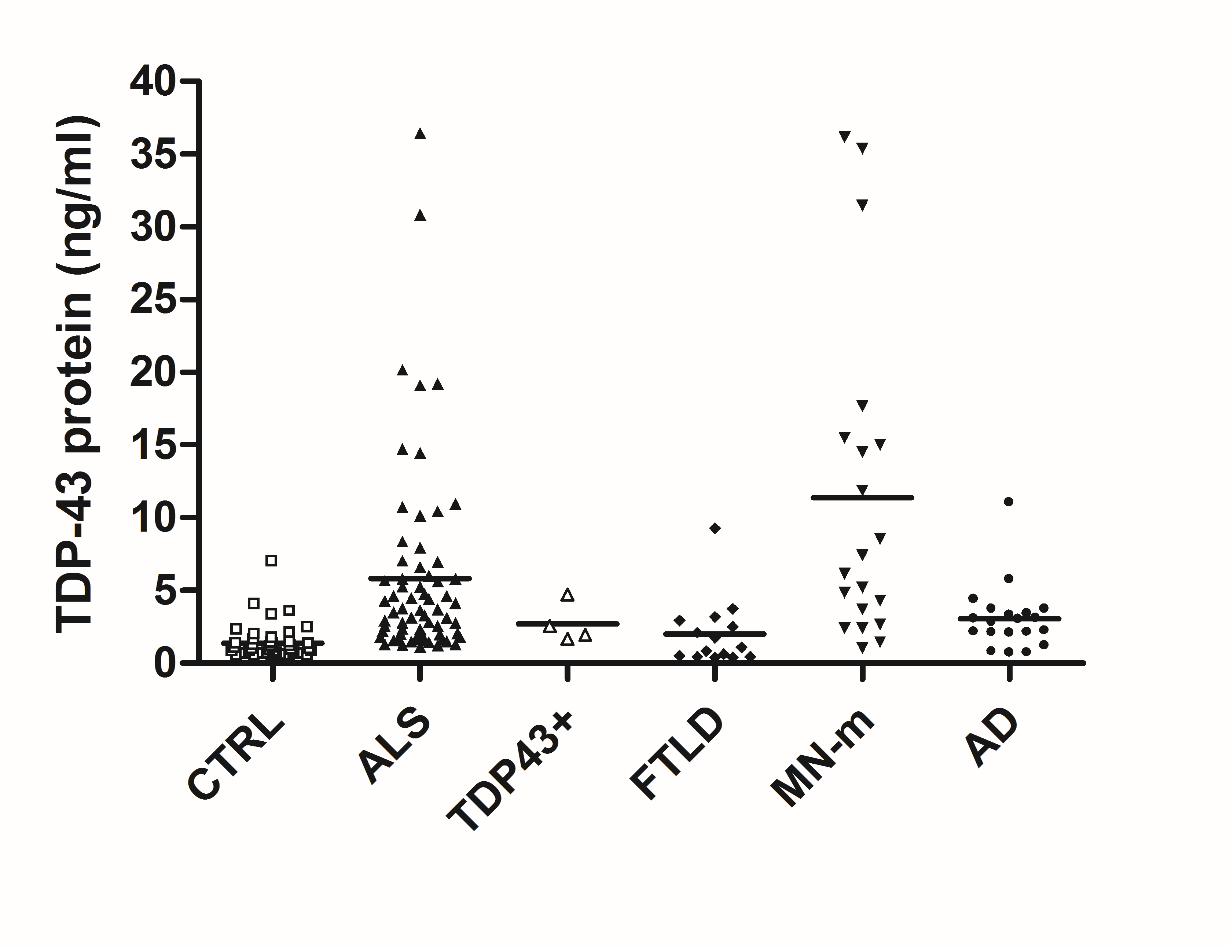

Supplement: Supplementary file 1 — Supplementary Figure 1. [file 41598_2021_81599_MOESM1_ESM.docx]
